# Fludarabine/TBI 8 Gy versus fludarabine/treosulfan conditioning in patients with AML in first complete remission: a study from the Acute Leukemia Working Party of the EBMT

**DOI:** 10.1038/s41409-023-01965-x

**Published:** 2023-03-31

**Authors:** Gesine Bug, Myriam Labopin, Riitta Niittyvuopio, Matthias Stelljes, Hans Christian Reinhardt, Inken Hilgendorf, Nicolaus Kröger, Ain Kaare, Wolfgang Bethge, Kerstin Schäfer-Eckart, Mareike Verbeek, Stephan Mielke, Kristina Carlson, Ali Bazarbachi, Alexandros Spyridonidis, Bipin N. Savani, Arnon Nagler, Mohamad Mohty

**Affiliations:** 1 Goethe University, Department of Medicine 2, Frankfurt am Main, Germany; 2 Sorbonne University, Department of Hematology, Saint Antoine Hospital, INSERM UMR 938, Paris, France; 3 HUCH Comprehensive Cancer Center, Stem Cell Transplantation Unit, Helsinki, Finland; 4 University of Muenster, Department of Hematology/Oncology, Muenster, Germany; 5 University Hospital Essen, Department of Hematology and Stem Cell Transplantation, Essen, Germany; 6 Universitätsklinikum Jena, Klinik für Innere Medizin II (Abt. Hämatologie und Onkologie) Am Klinikum 1, Jena, Germany; 7 Department for Stem Cell Transplantation, University Medical Center, Hamburg, Germany; 8 Tartu University Hospital, Clinic of Hematology and Oncology, Tartu, Estonia; 9 Universität Tuebingen, Medizinische Klinik, Tuebingen, Germany; 10 Klinikum Nuernberg, 5. Medizinische Klinik, BMT-Unit, Nuernberg, Germany; 11 Technical University of Munich, School of Medicine, Klinikum rechts der Isar, Clinic and Policlinic for Internal Medicine III, Munich, Germany; 12 Karolinska Institutet and University Hospital, LabMed, CAST, Karolinska Comprehensive Cancer Center, Stockholm, Sweden; 13 University Hospital, Department of Haematology, Uppsala, Sweden; 14 Bone marrow Transplant Program, Department of Internal Medicine, American University of Beirut Medical Center, Beirut, Lebanon; 15 Bone Marrow Transplantation Division, University Hospital Patras, Patras, Greece; 16 Long term Transplant Clinic, Vanderbilt University Medical Center, Nashville, TN USA; 17 Chaim Sheba Medical Center, Department of Bone Marrow Transplantation, Tel Hashomer, Israel

**Keywords:** Acute myeloid leukaemia, Stem-cell therapies

## Abstract

The optimal reduced intensity conditioning (RIC) regimen is a matter of debate. We retrospectively compared conditioning with fludarabine plus fractionated total body irradiation of 8 Gy (FluTBI) and fludarabine plus treosulfan 30, 36 or 42 g/m^2^ (FluTreo) in 754 patients with AML above the age of 40 years undergoing an allogeneic hematopoietic stem cell transplant (HSCT) in first complete remission (CR). After balancing patient characteristics by propensity score matching of 115 patients in each group, FluTBI was associated with a significantly lower probability of relapse compared to FluTreo (18.3% vs. 34.7%, *p* = 0.018) which was counteracted by a higher non-relapse mortality (NRM, 16.8% vs. 5.3%, *p* = 0.02). Thus, overall survival and graft-versus-host disease-free and relapse-free survival at 2 years were similar between groups (OS 66.9% vs. 67.8%, GRFS 50.3% vs. 45.6%). Univariate analysis by age group demonstrated a higher NRM exclusively in patients ≥55 years of age treated with FluTBI compared to FluTreo (27.6% vs. 5.8%, *p* = 0.02), while a similarly low NRM was observed in patients <55 years in both groups (6.0% vs. 4.7%, *p* = ns). We conclude that both conditioning regimens are effective and safe, but FluTBI may better be reserved for younger patients below the age of 55 years.

## Introduction

Allogeneic hematopoietic stem cell transplantation (HSCT) in first complete remission (CR) is the treatment of choice for the majority of patients with acute myeloid leukemia (AML) [1–3], but its antileukemic efficacy needs to be balanced against the risk of non-relapse morbidity and mortality [4]. To address the higher non-relapse mortality (NRM) associated with increasing age, especially with myeloablative conditioning (MAC) regimens, reduced intensity conditioning (RIC) has been widely adopted [5]. Unfortunately, RIC regimens may result in higher relapse rates especially in patients with measurable residual disease (MRD) as demonstrated in the large randomized BMT CTN 0901 trial [6, 7]. This trial compared a busulfan- or total body irradiation (TBI)-based MAC regimen to RIC consisting of fludarabine and an alkylating agent (i.e. IV busulfan 6.4 mg/kg, FB2 or melphalan ≤150 mg/m2, FluMel) in adults patients up to the age of 65 years with AML or myelodysplastic syndrome (MDS) and <5% bone marrow blasts at HSCT. Several efforts have been made to overcome the hurdle of insufficient antileukemic activity of RIC as well as excess tocixity of MAC regimens.

FB2 was challenged by fludarabine and treosulfan (FluTreo) in patients with AML in CR or MDS at increased risk of mortality with MAC. Treosulfan is a prodrug of a bifunctional alkylating agent with stem cell depleting and broad antileukemic activity and gained approval in the EU and Canada as part of conditioning based on a pivotal randomized trial in which FluTreo (30 g/m^2^ total dose) resulted in improved relapse-free survival (RFS) and OS (64% and 71% vs. 50% and 56% at 2 years, respectively), mainly due to reduction of late NRM from 22% to 11% [8]. In a randomized comparison of TBI-based MAC (i.e., fractionated TBI of 12 Gy and cyclophosphamide, TBI 12 Gy/Cy) with a reduced-toxicity conditioning (RTC) regimen of fludarabine and fractionated TBI of 8 Gy in AML in first CR (CR1), patients with AML aged 41 to 60 years treated with RTC achieved favorable RFS of 76% at 1 year and a sustained low NRM of 13% at 10 years [9, 10].

To date, no direct comparison of fludarabine and treosulfan (FluTreo) with fludarabine and TBI 8 Gy (FluTBI) has been performed. We hypothesize that FluTreo may be a lower toxicity alternative to the FluTBI 8 Gy regimen in AML patients aged above 40 years in CR1, who may not be prime candidates for MAC regimens.

## Methods

### Data collection

Data for this retrospective multicenter study were retrieved from the registry of the Acute Leukemia Working Party (ALWP) of the European Society for Blood and Marrow Transplantation (EBMT), a nonprofit, scientific society representing >600 transplant centers, mainly located in Europe. Centers commit to reporting all consecutive HSCT and follow-ups once a year. Data are entered, managed, and maintained in a central database and validated by verification of the computer printout of the entered data, cross-checking with the national registries, and on-site visits to selected teams. All patients gave informed consent authorizing the use of their personal information for research purposes. This study was approved by the ALWP of the EBMT institutional review board and conducted in accordance with the Declaration of Helsinki and Good Clinical Practice guidelines.

### Criteria for patient selection

Patients were included if they had (1) a diagnosis of AML in CR1 (MRD positive or negative); (2) an age >40 years; (3) received their first allogeneic HSCT between 2009 and 2019; (4) a matched sibling donor (MSD) or 10/10 HLA-matched unrelated donor (MUD), and if (5) peripheral blood stem cells or bone marrow was used as the stem cell graft; (6) the conditioning regimen consisted of either fludarabine and treosulfan (30, 36 or 42 g/m^2^, FluTreo) or fludarabine and fractionated TBI 8 Gy (4 × 2 Gy or 2 × 4 Gy, FluTBI). In vivo T- cell depletion (TCD) with anti-thymocyte globulin (ATG) was allowed, but transplantations from haploidentical donors, umbilical cord blood stem cells or using post-transplant cyclophosphamide or ex-vivo T-cell depletion were excluded. Transplant centers were asked to report MRD status at time of HSCT.

### Statistical analysis

The primary endpoint of this study was OS; secondary endpoints included LFS, cumulative incidence of relapse (CIR), NRM, incidence of acute and chronic graft-versus-host disease (GVHD) as well as survival free of grade III-IV acute GVHD or severe chronic GVHD (GRFS) [11]. Acute and chronic GVHD were diagnosed according to the modified Glucksberg criteria and modified Seattle criteria, respectively [12, 13].

Patient, disease, and transplant characteristics were compared by using the χ^2^ or Fisher’s exact test for categorial variables and the Mann-Whitney or Kruskal Wallis test for continuous variables. Probabilities for OS, LFS and GRFS were calculated using Kaplan-Meier estimates, and cumulative incidence (CI) curves for relapse, NRM, acute and chronic GVHD using a competing risk model: relapse and death are competing together, i.e. relapse is the competing event for NRM and death without relapse the competing event for relapse, whereas relapse and death were competing risks for GVHD [14]. Univariate analyses were performed using the log-rank test for LFS, OS, and GRFS, and Gray’s test for CI estimates [15].

For propensity score matching, exact matching was performed in a 1:1 ratio for donor type, secondary AML and adverse risk cytogenetics and nearest neighbor matching for age at HSCT, time from diagnosis to HSCT, female to male transplant, Karnofsky performance score (KPS) and in vivo TCD. We compared 115 patients in each conditioning group. All tests were two-sided with the type 1 error rate fixed at 0.05. SPSS 27.0 (IBM Corp., Armonk, NY, USA) and R 4.0.2 (R Core Team 2020. R: a language and environment for statistical computing. R Foundation for Statistical Computing, Vienna, Austria, https://www.Rproject.org/), were used for all statistical analyses.

## Results

### Patients and transplant procedures

A total of 754 patients with AML are included in this analysis, of whom 617 received FluTreo and 137 FluTBI conditioning. Patients in the FluTBI group were significantly younger with a median age of 53.7 (range, 40.1–70.7) vs. 60.7 (range 40.1–77.5) years. They had been transplanted a median of two years earlier (2014 vs. 2016) and had longer follow-up (median 45.9 vs. 26.9 months), Table 1. Additional statistically significant differences between the groups included a higher proportion of MSD with FluTBI (64.2% vs. 36.3%, *p* < 0.0001) as opposed to 10/10 MUD (35.8% vs. 63.7%, *p* < 0.0001), a shorter time from diagnosis to HSCT (3.8 (range, 1.8–16.2) vs. 4.7 (range, 1.7–22.9 months)) and a higher proportion of patients with de novo AML (84.7% vs. 76%). The groups did not differ significantly in terms of adverse risk cytogenetics according to European LeukemiaNet (ELN) 2017, patient or donor sex, KPS, or pre-transplant MRD status, although information on the latter parameter was available for only 287 patients.

**Table 1:** Patient, donor, and transplant characteristics according to conditioning regimen for all patients.

	FluTBI (*n* = 137)	FluTreo (*n* = 617)	*P*
Median patient age, years (range)	53.7 (40.1–70.7)	60.7 (40.1–77.5)	<0.0001
Karnofsky performance score			
<90%	27 (20.1%)	133 (22.2%)	0.60
≥90%	107 (79.9%)	465 (77.8%)	
Missing	3	19	
Diagnosis			
De novo AML	116 (84.7%)	469 (76%)	0.028
Secondary AML	21 (15.3%)	148 (24%)	
Cytogenetic risk group			
Good	4 (3.7%)	15 (3.4%)	0.074
Intermediate	75 (70.1%)	256 (58.9%)	
Poor	28 (26.2%)	164 (37.7%)	
NA/failed	30	182	
Not adverse	109 (79.6%)	453 (73.4%)	0.14
Adverse	28 (20.4%)	164 (26.6%)	
Median interval from diagnosis to HSCT, months (range)	3.8 (1.8–16.2)	4.7 (1.7–22.9)	<0.0001
Median year of HSCT (range)	2014 (2009–2019)	2016 (2009–2019)	<0.0001
MRD status pre-transplant			
MRD negative	31 (64.6%)	143 (59.8%)	0.54
MRD positive	17 (35.4%)	96 (40.2%)	
Missing	89	378	
Donor			
Matched sibling	88 (64.2%)	224 (36.3%)	<0.0001
10/10 HLA matched unrelated	49 (35.8%)	393 (63.7%)	
Patient sex			
Male	84 (61.3%)	329 (53.3%)	0.089
Female	53 (38.7%)	288 (46.7%)	
Donor/patient sex			
Female/male	26 (19%)	107 (17.4%)	0.66
Other combinations	111 (81%)	508 (82.6%)	
Missing	0	2	
Donor/patient CMV status			
Donor negative/patient negative	34 (26.0%)	109 (17.9%)	0.11
Donor positive/patient negative	11 (8.4%)	37 (6.1%)	
Donor negative/patient positive	30 (22.9%)	155 (25.6%)	
Donor positive/patient positive	56 (42.7%)	307 (50.5%)	
Missing	6	9	
TBI fractions		NA	
4 × 2 Gy	40 (29.2%)		
2 × 4 Gy	16 (11.7%)		
unknown	81 (59.1%)		
Treosulfan dose	NA		
3 × 10 g/m^2^		167 (27.1%)	
3 × 12 g/m^2^		122 (19.8%)	
3 × 14 g/m^2^		328 (53.2%)	
In vivo T-cell depletion			
No	74 (54%)	202 (32.7%)	<0.0001
ATG	63 (46%)	415 (67.3%)	
GvHD prevention			
Cyclosporin A + MTX	117 (85.4%)	425 (68.9%)	0.0003
Cyclosporin A + MMF	10 (7.3%)	124 (20.1%)	
Other	10 (7.3%)	68 (11.0%)	
Median follow-up, months [95% CI]	45.88 [35.83–56.96]	26.92 [24.19–31.03]	0.033

*HLA* human leukocyte antigen, *CMV* cytomegalovirus, *TBI* total body irradiation, *ATG* anti-thymocyte globulin, *MTX* methotrexate, *MMF* mycophenolate mofetil, *CI* confidence interval, *NA* not applicable.

The major difference in relation to transplant procedures was the significantly more frequent use of in vivo TCD with ATG in the FluTreo group (67.3% vs. 46%, *p* < 0.0001), Table 1.

### Pair-match analysis on propensity score

Because of the substantial difference in patient numbers between the two conditioning groups and significant differences in demographic and transplant-related parameters, we used propensity score matching to reduce the treatment assignment bias and create two patient groups of 115 each that were comparable for all observed covariates. Patient characteristics in the FluTBI and FluTreo group were well balanced in terms of age (median 55.2 vs. 54.9 years, KPS < 90% 22.6% and 23.5%, respectively), secondary AML (13% each), adverse cytogenetics (15.7% each), female donor to male recipient (19.1% vs. 17.4%) and time from diagnosis to HSCT (median 3.8 (range, 1.8–16.2) and 4.5 (range, 1.7–16.2) months, respectively), Table 2. An identical proportion of patients in both groups received grafts from MSD (61.7%) or 10/10 HLA-MUD (38.3%). In both groups, GVHD prophylaxis consisted predominantly of cyclosporin A (CSA) plus methotrexate (85.2% for FluTBI vs. 73.9% for FluTreo, p=ns), CSA and mycophenolate mofetil were given to 8.7% and 18.3% of patients in the FluTBI and FluTreo groups, respectively (p=ns). A similar proportion of patients in both groups (52.2% and 53.9%) received additional in vivo T-cell depletion with ATG.

**Table 2:** Patient, donor, and transplant characteristics according to conditioning regimen for patients included in the propensity score analysis.

	FluTBI (*n* = 115)	FluTreo (*n* = 115)	*P*
Median patient age, years (range)	55.2 (40.1–70.7)	54.9 (40.4–74.9)	0.96
Karnofsky performance score			
<90	26 (22.6%)	27 (23.5%)	0.88
>=90	89 (77.4%)	88 (76.5%)	
Diagnosis			
De novo AML	100 (87%)	100 (87%)	1
Secondary AML	15 (13%)	15 (13%)	
Cytogenetic risk group			
Good	4 (4.5%)	5 (6.5%)	
Intermediate	66 (75.0%)	54 (70.1%)	
Poor	18 (20.5%)	18 (23.4%)	
NA/failed	27	38	
Not adverse	97 (84.3%)	97 (84.3%)	1
Adverse	18 (15.7%)	18 (15.7%)	
FLT3 ITD			
Negative	65 (77.4%)	50 (67.6%)	0.17
Positive	19 (22.6%)	24 (32.4%)	
Missing	31	41	
NPM1			
Wildtype	64 (76.2%)	50 (67.6%)	0.39
Mutated	20 (23.8%)	21 (30%)	
Missing	31	45	
Median interval from diagnosis to HSCT, months (range)	3.8 (1.8–16.2)	4.5 (1.7–16.2)	0.15
Median year of HSCT (range)	2014 (2009–2019)	2016 (2009–2019)	0.005
MRD status pre-transplant			
MRD negative	37 (61.7%)	30 (58.8%)	0.76
MRD positive	23 (38.3%)	21 (41.2%)	
Missing	55	64	
Donor			
Matched sibling	71 (61.7%)	71 (61.7%)	1
10/10 HLA matched unrelated	44 (38.3%)	44 (38.3%)	
Patient sex			
Male	68 (59.1%)	64 (55.7%)	0.59
Female	47 (40.9%)	51 (44.3%)	
Donor/patient sex	70 (60.9%)	71 (61.7%)	0.89
Female/male	22 (19.1%)	20 (17.4%)	0.73
Other combinations	93 (80.9%)	95 (82.6%)	
Donor/patient CMV status			
Donor negative/patient positive	27 (24.3%)	28 (24.6%)	0.97
Other combinations	84 (75.7%)	86 (75.4%)	
Missing	4	1	
TBI fractions		NA	
4 × 2 Gy	33 (28.7%)		
2 × 4 Gy	15 (13.0%)		
unknown	67 (58.3%)		
Treosulfan dose	NA		
3 × 10 g/m^2^		27 (23.5%)	
3 × 12 g/m^2^		23 (20.0%)	
3 × 14 g/m^2^		65 (56.5%)	
In vivo T-cell depletion			
No	60 (52.2%)	62 (53.9%)	0.79
ATG	55 (47.8%)	53 (46.1%)	
GVHD prevention			
Cyclosporin A + MTX	98 (85.2%)	85 (73.9%)	0.08
Cyclosporin A + MMF	10 (8.7%)	21 (18.3%)	
Other	7 (6.1%)	9 (7.8%)	
Median follow-up, months [95% CI]	42.37 [31.52–53.77]	23.2 [20.44–32.74]	0.14

*HLA* human leukocyte antigen, *CMV* cytomegalovirus, *ATG* anti-thymocyte globulin, *TBI* total body irradiation, *MTX* methotrexate, *MMF* mycophenolate mofetil, *CI* confidence interval, *NA* not applicable.

**Fig. 1:**
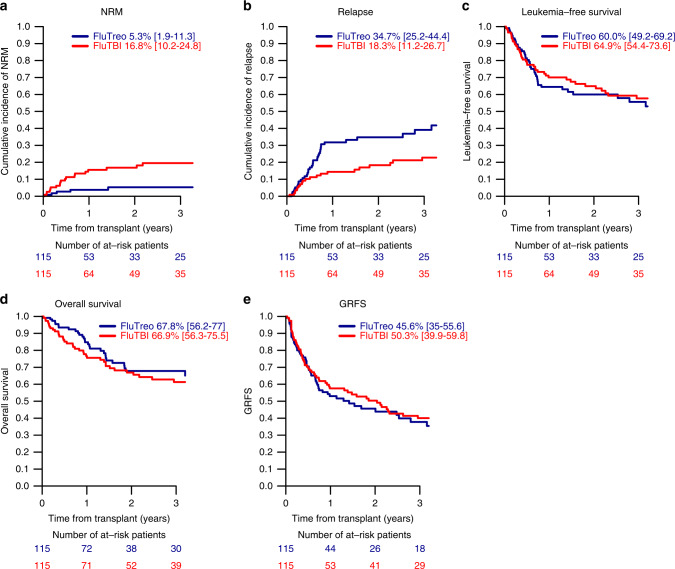
Comparison of FluTreo and FluTBI conditioning regimens. **a** Non-relapse mortality. **b** Relapse incidence. **c** Leukemia-free survival. **d** Overall survival. **e** Survival free of grade III-IV acute graft-versus-host disease (GVHD) or severe chronic GVHD. All results are at 2 years except acute GVHD at 180 days post-hematopoietic stem cell transplantation.

All but one patient in each group engrafted. Median follow-up of living patients was 42.4 months (range, 31.5–53.8) in the FluTBI and 23.2 months (range, 20.4–32.7) in the FluTreo group (*p* = 0.14). FluTBI was associated with a significantly lower CIR of 18.3% vs. 34.7% with FluTreo (*p* = 0.018, HR 0.51 (95% CI, 0.29–0.89)), but a higher NRM of 16.8% vs. 5.3%, *p* = 0.02, HR 3.0 (95% CI, 1.19–7.59), Fig. 1a, b. This difference in NRM was due exclusively to the higher NRM in patients ≥55 years of age (Table 3). LFS and OS were similar in the FluTBI and FluTreo groups (64.9% vs. 60.0%, HR 0.84 (95% CI, 0.54–1.31) and 66.9% vs. 67.8%, HR 1.08 (95% CI, 0.67–1.75)), respectively, Fig. 1c, d. Infection was the leading cause of death following FluTBI (*n* = 12, 34.3% vs. *n* = 3, 9.4% with FluTreo), whereas AML recurrence was the predominant cause of death in the FluTreo group (*n* = 15, 46.9% vs. *n* = 10, 28.6%). The frequency of death due to GVHD, multiorgan failure or interstitial pneumonitis did not differ between the two groups. Two patients developed a secondary malignancy after TBI conditioning, results not shown.

**Table 3:** Univariate analysis by age group for the pair-match analysis.

	FluTreo (*n* = 58)	FluTBI (*n* = 56)	FluTBI vs FluTreo HR (95% CI)	*p* (cluster = pair)
Age <55 years				
Relapse	33.6%[20.9–46.9]	20.5%[10.4–33]	0.51 (0.23–1.13)	0.10
NRM	4.7%[0.8–14.6]	6%[1.5–15]	1.68 (0.29–9.76)	0.57
LFS	61.7%[46.3–73.8]	73.5%[58.7–83.8]	0.62 (0.3–1.28)	0.19
OS	67%[50.1–79.3]	77.2%[62.4–86.7]	0.65 (0.3–1.42)	0.28
GRFS	46.6%[31.4–60.4]	59.7%[44.6–72]	0.69 (0.41–1.18)	0.18
Acute GVHD II-IV	21.4%[11.8–33]	20.1%[10.7–31.7]	0.89 (0.37–2.14)	0.79
Acute GVHD III-IV	5.4%[1.4–13.5]	7.3%[2.3–16.3]	1.28 (0.28–5.72)	0.75
Chronic GVHD	54.7%[38.3–68.4]	35.7%[22.1–49.5]	0.51 (0.29–0.93)	0.027
Extensive chronic GVHD	19.6%[8.8–33.6]	10.7%[3.8–21.6]	0.64 (0.24–1.74)	0.39
	**FluTreo (*n*** = **57)**	**FluTBI (*n*** = **59)**	**FluTBI vs FluTreo HR (95% CI)**	***p*** **(cluster** = **pair)**
Age ≥55 years				
Relapse	35.7%[21.9–49.8]	16.3%[7.3–28.5]	0.52 (0.24–1.13)	0.10
NRM	5.8%[1.5–14.7]	27.6%[15.9–40.7]	3.74 (1.23–11.43)	**0.02**
LFS	58.4%[42.7–71.3]	56%[40.6–68.9]	1.09 (0.62–1.91)	0.78
OS	68.7%[51.6–80.9]	56.2%[40.5–69.3]	1.67 (0.89–3.15)	0.11
GRFS	45%[30.1–58.8]	40.4%[26.2–54.2]	0.98 (0.62–1.55)	0.92
Acute GVHD II-IV	20%[10.6–31.5]	25.4%[15.1–37.1]	1.21 (0.57–2.59)	0.62
Acute GVHD III-IV	12.7%[5.5–23]	5.1%[1.3–12.9]	0.34 (0.09–1.31)	0.12
Chronic GVHD	39%[24.3–53.4]	49.5%[34.4–63]	1.31 (0.78–2.21)	0.3
Extensive chronic GVHD	19.7%[8.9–33.5]	22.7%[12–35.5]	0.91 (0.45–1.82)	0.78

All results are at 2 years except acute GVHD at 180 days post HSCT.

*NRM* non-relapse mortality, *LFS* leukemia-free survival, *OS* overall survival, *GVHD* graft-versus-host disease, *GRFS* survival free of grade III-IV acute GVHD or severe chronic GVHD.

There was no statistically significant difference between FluTBI and FluTreo in the CI of acute GVHD II-IV (22.8% vs. 20.7%, HR 1.05), GVHD III-IV (6.2% vs. 9.0%, HR 0.59), chronic GVHD (42.6% vs. 47.5%, HR 0.81) or extensive chronic GVHD (16.8% vs. 19.6%, HR 0.76), results not shown, resulting in similar GRFS of 50.3% and 45.6%, HR 0.83, respectively (Fig. 1e).

## Discussion

Because of its manageable extramedullary toxicity profile and satisfactory anti-leukemic activity in a randomized registration study, the combination of fludarabine and treosulfan 30 g/m^2^ has been increasingly adopted as the RIC of choice in patients with AML and MDS who are ineligible for MAC [8]. In a large retrospective study, this conditioning regimen using higher treosulfan doses was shown to be tolerable and effective in patients with more advanced AML and a median age of 57 years [16]. We hypothesized that FluTreo might be an alternative to the RTC FluTBI 8 Gy in patients above 40 years with AML in CR1, for whom favorable long-term outcomes have recently been reported in a randomized comparison with TBI 12 Gy/Cy MAC [9, 10]. Our study demonstrates that FluTBI conditioning prior to 10/10 HLA matched allogeneic HSCT achieves good leukemic control in this patient population with a low relapse rate of 18.3% and modest NRM of 16.8%. The probabilities of LFS and OS at two years (64.9% and 66.9%, respectively) match the outcome data reported for the subgroup of patients aged 41–60 years in the FluTBI 8 Gy arm of the German MAC vs. RTC trial.

Our hypothesis that the antileukemic efficacy of FluTreo would be equivalent to that of FluTBI was not borne out by the results of our pair-match analysis, which demonstrated a significantly higher CIR with FluTreo compared with FluTBI conditioning. Moreover, the relapse rate of 34.7% in our FluTreo cohort was higher than in the FluTreo arm (24.6%) of the randomized registration trial [8]. This may be attributable to differences in the patient population, as the latter study included not only AML, but also 30% of MDS patients, with MDS patients experiencing fewer relapses (Supplementary Appendix [8],). In addition, a larger proportion of patients in the randomized study developed chronic, and in particular mild chronic GVHD, which may have contributed to the lower relapse rate. As chronic GVHD did not significantly contribute to mortality in either study, the lower incidence of chronic GVHD seen in our analysis is consistent with the higher observed CIR. In vivo TCD was included in the propensity score for pair-matching and its distribution well balanced between the two conditioning regimens (47.8% vs 46.1% in FluTBI and FluTreo groups, respectively). Consequently, it cannot explain the higher risk of relapse in the FluTreo group.

An additional possibility is that differences in the treosulfan dose may have contributed to the unexpectedly high relapse rate although there is no conclusive evidence of greater antileukemic efficacy of higher treosulfan doses. NRM however proved to be higher with 42 g/m^2^ compared to the 30 g/m^2^ dose in the pivotal randomized study which was stopped after an interim analysis had shown prolonged neutropenia and subsequent serious infectious complications with fludarabine and treosulfan 42 g/m^2^ total dose compared to the RIC regimen FB2. The protocol was amended to a reduced treosulfan dose of 30 g/m2 and demonstrated superior overall and relapse-free survival of patients in the FluTreo arm [8, 17]. In our study, NRM was low despite most patients receiving treosulfan doses higher than 30 g/m^2^.

Comparing the patient cohort in our analysis and the randomized study evaluating FluTBI 8 Gy, NRM in the FluTBI group in our study seemed to be somewhat higher, with the caveat of a different duration of follow-up (16.8% at 2 years vs. 13.0% at 10 years). We show that a 55 year age threshold discriminates between patients with low and high NRM, even though this does not translate into inferior OS and RFS in the older age cohort. This discrepancy may be due to the fact that the lower relapse rate with FluTBI did not reach statistical significance given the relatively small number of patients. Nevertheless, it appears advisable to employ FluTBI with considerable caution in patients 55 years and above. Another possible explanation for this difference in NRM is the TBI schedule used in these two studies: whereas the randomized study consistently administered TBI in four fractions of 2 Gy, our analysis also included patients in whom 8 Gy TBI was delivered in 2 fractions of 4 Gy. A retrospective study comparing delivery of 12 Gy TBI in one or two fractions over 3 days suggested a higher risk of organ toxicity, but not NRM with the 1-day fractionation [18]. Additional confounding variables might have been introduced by the heterogeneity of TBI techniques used in different centers [19] and/or by center preferences in the application of only FluTBI (8 centers), only FluTreo (52 centers) or both (15 centers). However, we found no such center effects (data not shown) [20].

In addition to age, the level of MRD at the time of HSCT is a well-known determinant of relapse rate and outcome. There was no significant difference in the proportion of MRD positive and negative patients in the two conditioning groups in our study. However, MRD levels at transplant were available for only a minority of patients and techniques of MRD detection heterogeneous among centers [21], which is a limitation of the present analysis. After HSCT, incomplete T cell donor chimerism may identify AML patients at high risk for disease recurrence, but this information has not been captured in the EBMT registry [22].

Taken together, our retrospective analysis demonstrates that the FluTBI and FluTreo conditioning regimens result in comparable survival in patients with AML undergoing HSCT in CR1. In view of the randomized BMT CTN 0901 trial, which was reported after the time period encompassing our present analysis, centers may prefer a MAC regimen including TBI 12 Gy or busulfan 12.8 mg/kg for patients who proceed to HSCT with MRD positive AML [6, 7]. However, at least the RTC FluTBI evaluated in our study is more intensive than the RIC regimens explored in the BMT CTN 0901 trial [23], and our study furthermore includes a separate analysis of NRM in patients <55 and ≥55 years of age. In the latter patients, the more intensive FluTBI regimen was associated with a significantly higher NRM, whereas the age-dependency of NRM was not analysed separately in the BMT CTN 0901 trial. Although we did not identify patient subgroups who derived significant benefit from the enhanced antileukemic activity of FluTBI, patients <55 years of age with high-risk leukemias and a low HCT-specific comorbidity index (HCT-CI) may do better with FluTBI. Robust long-term outcome data consistent with this concept have been reported [10].

NRM with FluTreo was remarkably low even in patients at higher risk of toxic death and could likely be the preferred type of conditioning for patients with such risk features [5]. It will also be of interest to determine whether the more user friendly chemotherapy based FluTreo regimen may have fewer long-term side effects than TBI based conditioning, provided outcome is the same. A similarly favorable NRM was reported in a randomized trial for the widely used RIC regimen fludarabine and TBI 2 Gy although for the price of a less efficacious disease control in a variety of hematologic diseases compared to FB2 [24]. In an effort to further enhance the antileukemic activity of FB2, the augmented conditioning regimen FLAMSA-Bu was applied to patients with high-risk AML in CR1, CR2 or with primary refractory disease in the randomized FIGARO trial but failed to improve relapse rates compared to the RIC FluMel [25].

Our results strongly suggest that strategies that build on the excellent tolerability of FluTreo and focus on reducing the higher relapse rate associated with this regimen conceptually have promise. Recently, O´Hagan Henderson et al. demonstrated the feasibility of fludarabine and treosulfan 42 g/m^2^ in combination with high-dose cytarabine in 77 patients with poor-risk myeloid neoplasms, 54% of whom were not in CR. In the subgroup of 58 AML patients, OS and CIR at 3 years were 44% and 43%, respectively [26]. The combination of FluTreo with TBI 2 Gy has been pioneered by Gyurkocza et al. in AML and MDS patients [27], analogous to the successful sequential conditioning regimen of fludarabine and melphalan followed by TBI 8 Gy in relapsed and refractory AML [28]. Addition of low-dose TBI to FluTreo was associated with considerable gastrointestinal toxicity but nevertheless a low NRM of 8% and a CIR of 27% at 2 years [27]. Conspicuously, this approach did not appear to mitigate the high post-transplant relapse rate in patients with MRD pre-HSCT as opposed to patients who were MRD negative (CIR 70% and 18%, respectively). This highlights the need to also explore additional approaches such as post-transplant maintenance strategies and more effective pre-transplant therapies.

## Supplementary information

Principal investigators of the contributing institutions

## Data Availability

Upon request to the ALWP of the EBMT (Dr Myriam Labopin).
