# Supplementary material for: Fludarabine/TBI 8 Gy versus fludarabine/treosulfan conditioning in patients with AML in first complete remission: a study from the Acute Leukemia Working Party of the EBMT
Source: Bone Marrow Transplant. 2023 Mar 31;58(6):710–6. doi: 10.1038/s41409-023-01965-x (PMC10247361; doi:10.1038/s41409-023-01965-x)
Supplement: Supplementary file 1 — Principal investigators of the contributing institutions [file 41409_2023_1965_MOESM1_ESM.docx]

Supplementary Information: Principal investigators of the contributing institutions

Tobias Gedde-Dahl, Oslo University Hospital, Rikshospitalet, Clinic for Cancer Medicine, Hematology Dept., Section for Stem Cell Transplantation, Oslo, Norway. Peter Dreger, University of Heidelberg, Medizinische Klinik u. Poliklinik V, Heidelberg, Germany. Edgar Jost, University Hospital Aachen, Dept. of Oncology, Hematology and SCT, Medizinische Klinik IV, Aachen, Germany. Martin Kaufmann, Robert-Bosch-Krankenhaus, Abt. Hämatologie / Onkologie, Stuttgart, Germany. Sebastian Giebel, Department of Bone Marrow Transplantation and Oncohematology, Maria Sklodowska-Curie National Research Institute of Oncology, Oncology Center, Gliwice Branch, Gliwice, Poland. Ioanna Sakellari, George Papanicolaou General Hospital, Haematology Department / BMT Unit, Thessaloniki, Greece. Martin Bornhäuser, Universitaetsklinikum Dresden, Medizinische Klinik und Poliklinik I, Dresden, Germany. Jochen Casper, Klinikum Oldenburg, Abt. Onkologie/Hämatologie, Oldenburg, Germany. Henrik Sengeloev, Bone Marrow Transplant Unit L 4043, National University Hospital, Rigshospitalet, Copenhagen, Denmark. Fabio Ciceri, Ospedale San Raffaele s.r.l., Haematology and BMT, Milano, Italy. Renato Fanin, Azienda Ospedaliero Universitaria di Udine, Division of Hematology, Udine, Italy. Christoph Schmid, Klinikum Augsburg, II Medizinische Klinik, Augsburg, Germany. Péter Reményi, Dél-pesti Centrumkórház, Országos Hematológiai és Infektológiai Intézet, Dept. Haematology and Stem Cell Transplant, Budapest, Hungary. Andreas Neubauer, Philipps Universitaet Marburg, University Hospital Giessen and Marburg, Marburg, Germany. Cecilia Isaksson, Umea University Hospital, Hematology, Umea, Sweden. Maija Itäla-Remes, Turku University Hospital, TD7 (Stem Cell Transplant Unit), Turku, Finland. Yves Chalandon, Département d`Oncologie, Service d`Hématologie, Hôpitaux Universitaires De Genève, Geneva, Switzerland. Harald Biersack, University Medical Center Schleswig-Holstein, Clinic for Hematology/Oncology and Stem cell transplantation, Luebeck, Germany. Igor Wolfgang Blau, Medizinische Klinik m. S. Hämatologie, Onkologie und Tumorimmunologie, Charité Universitätsmedizin Berlin, Berlin, Germany. Domenico Russo, USD Trapianti di Midollo, Adulti, Universita di Brescia, Brescia, Italy. Guido Kobbe, Heinrich Heine Universitaet, Klinik für Hämat., Onkol., Klin. Immun., Duesseldorf, Germany. Corrado Tarella, European Institute of Oncology, Institute of Haematology, Milano, Italy. Tsila Zuckerman, Rambam Medical Center, Dept. of Hematology & BMT, Haifa, Israel. Marieke Dürholt, Evangelisches Krankenhaus Essen-Werden gGmbH, Haematologie/Onkologie/Stammzelltransplantation, Essen, Germany. Lorenz Thurner, University of Saarland, University Hospital, Dept. of Internal Med., BMT Unit, Homburg, Germany. Stefan Klein, Universitaetsmedizin Mannheim, III. Medizinische Klinik, Einheit für Stammzelltransplantation, Mannheim, Germany. Aymen Bushra Ahmed, Haukeland University Hospital, Department of Haematology, Bergen, Norway. Didier Blaise, Programme de Transplantation & Therapie Cellulaire, Centre de Recherche en Cancérologie de Marseille, Institut Paoli Calmettes, Marseille, France. Lutz Peter Müller, Martin-Luther-Universitaet Halle-Wittenberg, Klinik für Innere Medizin IV, Halle, Germany. Giorgio La Nasa, Centro Trapianti Unico Di CSE Adulti e Pediatrico A. O Brotzu, Italy. Roland Schroers, ZSIS Universitaetsklinikum Knappschaftskrankenhaus Bochum GmbH, Medizinische Klinik - Hämatologie und Onkologie, Bochum, Germany. Régis Peffault de Latour, Hopital St. Louis, Dept.of Hematology - BMT, Paris, France. Edouard Forcade, CHU Bordeaux, Hôpital Haut-leveque, Pessac, France. Arne Brecht, Deutsche Klinik fuer Diagnostik, KMT Zentrum, Wiesbaden, Germany. Uwe Platzbecker, Medical Clinic and Policinic 1, Hematology and Cellular Therapy, University hospital Leipzig, Leipzig, Germany. Judith Niederland, HELIOS Klinikum Berlin-Buch, Klinik für Hämatologie und Stammzelltransplantation, Berlin, Germany. Hélène Labussière-Wallet, Centre Hospitalier Lyon Sud, Pavillon Marcel Bérard -Bat 1G, Service Hematologie, Lyon, France. Herman Einsele, Universitaetsklinikum Wuerzburg, Med. Klinik und Poliklinik II, Wuerzburg, Germany. Arancha Bermúdez Rodríguez, Hospital U. Marqués de Valdecilla, Servicio de Hematología-Hemoterapia, Santander, Spain. Arnold Ganser, Hannover Medical School, Department of Haematology, Hemostasis, Oncology, and Stem Cell Transplantation, Hannover, Germany. Thomas Heinicke, Universitaetsklinium Magdeburg, Med. Fakultät d., Klin.f.Hämatol./Onkologie, Magdeburg, Germany. Johanna Tischer, Klinikum Grosshadern, Med. Klinik III, Marchioninistr. 15, Munich, Germany. William Krüger, Klinik fuer Innere Medizin C, Hämatologie und Onkologie, Transplantationszentrum, Palliativmedizin, Universitätsmedizin Greifswald, Greifswald, Germany. Angela Cuoghi, Azienda Ospedaliero Universitaria di Modena Policlinico, Ematologia, Modena, Italy. Alessandro Rambaldi, ASST Papa Giovanni XXIII, Hematology and Bone Marrow Transplant Unit, Bergamo, Italy. Matthias Edinger, University Regensburg, Dept. of Hematology and Oncology, Regensburg, Germany. Wolf Rösler, University Hospital Erlangen, Dept. of Internal Medicine 5, Erlangen, Germany. Nadezda Basara, St. Franziskus Hospital, Medizinische Klinik I, Flensburg, Germany. Michael Kiehl, Klinikum Frankfurt (Oder) GmbH, Medizinische Klinik I, Frankfurt-Oder, Germany. Xavier Poiré, Cliniques Universitaires St. Luc, Dept. of Haematology, Brussels, Belgium. Polina Stepensky, Hadassah University Hospital, Dept. of Bone Marrow Transplantation, Jerusalem, Israel. Stig Lenhoff, Skanes University Hospital, Dept. of Hematology, Lund, Sweden. Jan-Erik Johansson, Sahlgrenska University Hospital, Center for Hematopoietic Cell Transplantation, Hematology Section, Goeteborg, Sweden. Marco Casini, Hospital San Maurizio, Dept. of Hematology - BMT Unit, Bolzano, Italy. Christof Scheid, University of Cologne, I. Dept. of Medicine, Cologne, Germany. Axel A. Fauser, Klinik fuer Knochenmarktransplantation, und Hämatologie/Onkologie GmbH, Idar-Oberstein, Germany. Gitte Olesen, Aaruhus University Hospital, Aarhus Amtssygehus, Aarhus, Denmark. Jan Vydra, Institute of Hematology and Blood Transfusion, Servicio de Hematología, Prague, Czech Republic. Anna Bergendahl Sandstedt, University Hospital, Dept. of Hematology, Linkoeping, Sweden. Ipek Yonal-Hindilerden, Ýstanbul Tip Fakultesi, Iç Hastaliklari ABD, Kemik iliði nakil unitesi, Istanbul, Turkey. Burak Deveci, Medstar Antalya Hospital, Stem Cell Transplantation Unit, Cakirlar, Antalya, Turkey
